# Evaluating mechanisms that could support credible reputations and cooperation: cross-checking and social bonding

**DOI:** 10.1098/rstb.2020.0302

**Published:** 2021-11-22

**Authors:** Flóra Samu, Károly Takács

**Affiliations:** ^1^ The Institute for Analytical Sociology, Linköping University, Norrköping, Sweden; ^2^ Doctoral School of Sociology, Corvinus University of Budapest, Budapest, Hungary; ^3^ Centre for Economic and Regional Studies, Agglomeration and Social Networks Lendület Research Group, Budapest, Hungary; ^4^ Centre for Social Sciences (TK), Computational Social Sciences – Research Center for Educational and Network Studies (CSS-RECENS), Budapest, Hungary

**Keywords:** gossip, Prisoner's Dilemma, reputation, social bonding, cross-checking

## Abstract

Gossip is believed to be an informal device that alleviates the problem of cooperation in humans. Communication about previous acts and passing on reputational information could be valuable for conditional action in cooperation problems and pose a punishment threat to defectors. It is an open question, however, what kind of mechanisms can make gossip honest and credible and reputational information reliable, especially if intense competition for reputations does not exclusively dictate passing on honest information. We propose two mechanisms that could support the honesty and credibility of gossip under such a conflict of interest. One is the possibility of voluntary checks of received evaluative information from different sources and the other is social bonding between the sender and the receiver. We tested the efficiency of cross-checking and social bonding in a laboratory experiment where subjects played the Prisoner's Dilemma with gossip interactions. Although individuals had confidence in gossip in both conditions, we found that, overall, neither the opportunities for cross-checking nor bonding were able to maintain cooperation. Meanwhile, strong competition for reputation increased cooperation when individuals' payoffs depended greatly on their position relative to their rivals. Our results suggest that intense competition for reputation facilitates gossip functioning as an informal device promoting cooperation.

This article is part of the theme issue ‘The language of cooperation: reputation and honest signalling’.

## 1. Introduction

The problem of cooperation has received multidisciplinary attention (see [1–4] for review) due to its prevalence for a variety of contexts in life. As individual interests work against cooperation, it is a puzzle why cooperation is observed at all, particularly among individuals who are not related to each other and are not engaged in repeated interaction. For such situations, indirect reciprocity has been proposed as a solution [5–8]. It has been suggested that humans have been able to solve the problem of cooperation beyond repeated encounters in small groups because they could rely on informal tools that facilitated the efficiency of downstream indirect reciprocity mechanisms [9,10]. Gossip is believed to be such an informal tool that enables cooperation as it transmits key information about third parties who are potential interaction partners and hence facilitates the selection of cooperative choice against partners who have good reputation [11–14]. Gossip may stem from sanctioning motives by which individuals can punish or pose a threat to individuals who were about to exploit cooperation efforts [15–18]. The alleged relationship between gossip and cooperation through the construction of reputations has received empirical support in laboratory experiments [19–24].

Explanations that link gossip to cooperation are valid only if we assume that gossip contains real information and negative gossip targets those individuals who attempted to exploit cooperation efforts. Gossip, however, is not necessarily honest and credible [25–28]. Distortion might occur from misinterpretation of actions (cf. using first-order social norms, [29,30]), but it could also be the result of strategic manipulation by the sender [31].

Once gossip is not in line with actions, reputations on which individuals base their decisions become unreliable, so over time, they lose information value. As a consequence, cooperation collapses if it is built up on the shaky ground of miscredited gossip [32,33]. Therefore, how gossip could help establish cooperation needs a more thorough investigation. For this purpose, we need to be aware of mechanisms that can maintain the credibility of gossip reputations and we need to know if reliable reputations are sufficient for the maintenance of cooperation. We propose three mechanisms that might be linked to honest gossip, reliable reputations and could undermine or empower cooperation conditional on reputational information.

### (a) Competition decreases the reliability of gossip

The transmission of reputational information might not be honest due to the conflict of interest between the sender and the target. Competition for profitable partners [34,35], for social status [36,37] or for reputation-related benefits [24] could all create conflicts of interest. Regardless of the ultimate goal, a good reputation is the target of the competition itself for which both the sender and the target are competing. If reputation is a restricted good, then the conflict of interest might more likely be realized and taken into consideration in communication decisions.

Accordingly, the strive for good reputation drives not only generosity [38–42], but as an alternative tool for individuals to improve their relative rank, also dishonest gossip about rivals [43–45]. Unlike random noise [46] and exaggeration [23], once such strategic misrepresentations are of a realistic possibility, the reliability of social information exchange could be questioned [47] and the alleged link between gossip and cooperation is broken [48]. In previous experiments, dishonesty was brought about by competition between the sender and receiver of gossip [48], but it has not been tested whether people will mislead their audience with dishonest information if they have a conflict of interest only with the target. We investigate how competition for reputational benefit contributes to the greater presence of dishonest gossip signals and indirectly, how this possible strategic misrepresentation affects reputation-based cooperation.

### (b) Cross-checking increases the reliability of gossip

Individuals actively seek social information to condition their future actions on a better-informed ground [49]. If the same evaluative content is received from multiple sources, then the reliability of gossip increases [50]. As the number of sources increases, dishonesty may be deterred [51,52], since it can be better discovered [53], possibly implying a cost for the sender [54]. There is no agreement in the literature if multiple sources should be independent in order to channel in information from diverse sources [55,56] or they should rather originate from trusted and well-embedded sources from the local network [57]. It is known, however, that in an unstructured information regime, more gossip better facilitates individual inclinations towards cooperation [20].

Previously, complete information about partners’ previous behaviour was condensed in gossip statements and an empirical study on the effect of multiple but uncertain gossip on reputation is still a ‘missing piece’ [20, p. 2534]. In this study, we address this gap by testing the effect of cross-checking by multiple sources on the reliability of gossip.

### (c) Social bonding increases the reliability of gossip

Gossip is certainly more than just a form of informal punishment or a deterrence device to avoid free riding. It has been shown that gossip could harmonize the relationship between the sender and the receiver and strengthen their social bonding [58]. This way, gossip has a similar affiliative impact among humans [59,60] as social interactions in other species such as social play [61], sensitive touch [62], food sharing [63], gestural modality [64] and grooming [65–67], which provide necessary preconditions for cooperation in a situation with conflict, such as mobilization against external or internal threats. More attention to prosocial norms, and mutual expectations about corresponding behaviour, which develop unconsciously as a result of informal communication, can contribute to higher commitment to cooperation [68–70].

Beyond the role of gossip in unconscious bonding, people can also consciously use gossip to form partnerships [71]. We argue that social bonds are created through gossip only if social information is honest, because dishonesty decreases the reputation of the sender [72] and only honest reputational information can lead to a trusted relationship [73,74]. In this study, we examine the extent to which the two proposed corrective mechanisms (cross-checking and social bonding) can mitigate the potential negative impact of competition.

## 2. Methods

### (a) Participants

Two hundred and thirty-four students of the Corvinus University of Budapest participated in a laboratory experiment between January and May 2019. The call was advertised through the university e-mail system and any interested person was able to apply for the experiment through a separate recruitment interface. After arrival to the laboratory, instructions were displayed on participants' screens and were distributed in hard copy as well. Processing of the instructions was tested with questions. Players participated in the experiment anonymously. In order to make participants traceable during the experiment, we identified them with names of planets’ moons. All names started with different letters of the alphabet to assist memory capacities. The experiment lasted for an average of 45 min, and it took an average of 10 min to complete the questionnaire following the experiment. The final profit was calculated as the average payoff of six randomly selected rounds. In addition to the final payoff, a show-up fee (HUF 1000) had been paid to the participants. The average payoff was HUF 1807 (approx. 5 EUR). The experiment was programmed with z-Tree [75].

### (b) Design

We manipulated (1) the level of competition and (2) the presence of mechanisms that can maintain the credibility of gossip (cross-checking, and social bonding) in our experiment between sessions. We introduced competition to increase the likelihood of dishonest gossiping and test whether cross-checking and social bonding mechanisms can eliminate incentivized dishonesty about rivals. Therefore, we interacted manipulation 1 with manipulation 2. With a control condition in which neither cross-checking nor social bonding opportunities were present, we obtained a 2 (competition: high, low) × 3 (mechanism for credible gossip: control, cross-checking, social bonding) factorial design.

Each possible treatment was played in two sessions, so we organized a total of 12 sessions. We had 20 participants in 10 out of the 12 sessions. Eighteen were present in one (low competition—control) and 16 in another session (high competition—cross-checking).

**Figure 1.**
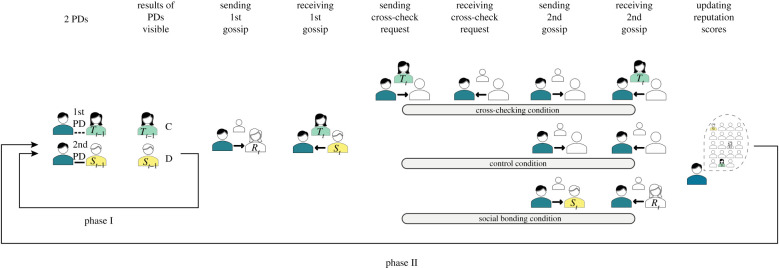
Steps of the experiment within one round. Each round starts with a Prisoner's Dilemma (2 PDs) game, followed by gossip exchange according to treatments and the assessment of other participants' trustworthiness (updating reputation scores). (Online version in colour.)

The experiment was divided into two phases. The first phase covered the first five rounds, the second phase lasted from round six until the end of the experiment. Participants did not know when the experiment would end. In the first phase, participants played a Prisoner's Dilemma game (PD); in the second phase, in addition to the PD, they had the opportunity to gossip and evaluate others (figure 1).

### (c) Procedure

#### (i) Phase I: basic level of cooperation without communication

At the beginning of each round, individuals were randomly paired with two other players whose fictitious names appeared on the screen and played separate two-person PD games with them (see translated screen 1 in electronic supplementary material, S2). Neutral framing was used in the experiment: options were labelled with letters (*L* and *R*). Outcomes were set as follows. If both players cooperated, they earned HUF 1500 (*R*); in contrast, if both defected, they received HUF 500 (*P*). A person who cooperated while the partner defected was not entitled to payment (*S*). Conversely, the partner's payment was HUF 2000 (*T*). The payoff structure was calibrated such that the index of cooperation ([76,77]; (*R* − *P*)/(*T* − *S*) = 0.5) shows a moderate conflict between self- and group interest. Participants had 23 s to decide in the two PD games. If players ran out of time, they got HUF 0, and their PD partner's payoffs depended on their decisions (HUF 0 after cooperation, HUF 500 after defection). In this regard, running out of time was equivalent to defection (cf. [78]), so it could not be used as a costly punishment action. In the first round, 63 players (26.9%) ran out of time, and 44 (18.8%) in the sixth round. Outside these introductory rounds, typically 1–2 people (*M* = 1.52) did not decide in time. Participants saw the results of their own games on the subsequent screen (see screen 2 in electronic supplementary material, S2).

#### (ii) Phase II: the reliability of gossip and its effect on cooperation

In the second phase of the experiment, participants played the same PD games as before. In addition, changes were introduced regarding gossip opportunities and reputation building. After the PD game, gossip could be sent to a randomly selected participant (figure 1 or screen 3 in electronic supplementary material, S2). In each round, participants could send two messages. The fictitious names of gossip targets and receivers were displayed on the screen. Participants could select the valence of gossip from three options indicated by happy, neutral and sad emoticons. We have employed emoticons as they simplify and clarify the content of reputation scores and translate evaluations into positive, neutral or negative judgement. Sending gossip was free and optional and was possible within a limit of 18 s. After the first gossip message, we manipulated how the second message proceeded (figure 1 and section about manipulation 2).

**Figure 2.**
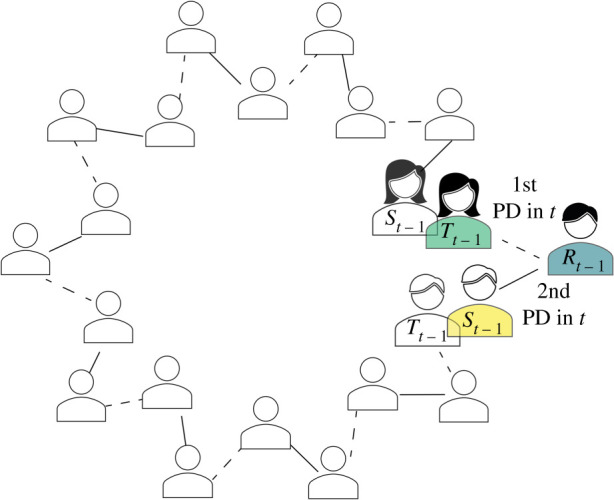
PD partner matching. In the second phase of the experiment, in each round, players were drawn into one of the two roles that determine who they play the PD game in that round with: (i) half of the participants (outer circle, *R*) played one PD with their first gossip source in the previous round (*S_t_*_−1_) and one PD with a target of the gossip from this source (*T_t_*_−1_); and (ii) the other half of the participants (inner circle, *S* and *T* ), accordingly, played one PD (solid line) with a receiver of the gossip sent by them (*R_t_*_−1_, for *S_t_*_−1_, not tagged for *T_t_*_−1_) and one PD (dashed line) with a participant who received gossip about them (*R_t_*_−1_, for *T_t_*_−1_, not tagged for *S_t_*_−1_). (Online version in colour.)

After the first round in phase II (round 7), players played one of the PDs with their gossip partner from the previous round. The other PD partner was the target of gossip from the previous round. To control who is playing with whom in the next round, the target of the gossip was randomly selected. In one round, only half of the matching resulted in PDs with previous gossip senders and targets. The inverse rule has been applied to the other half of the participants: they played with the receiver of their first gossip and who received a message about them (figure 2). The computer determined randomly who belongs to which half at the beginning of each round. Players were aware of these matching rules.

From round 6, besides gossip, players could assign reputation scores to other participants. They were asked to evaluate on a scale of 0–100 according to how much they ‘trust other participants'. These individually assigned private reputation scores are hence not consensual. In round 6, everyone's score was set to a starting value of 50, but changes were saved to subsequent rounds, thus, players were able to use the saved reputation scores they assigned. Fifty seconds were available for the assignment of reputation scores. Each round ended with a summary where players learned their own average reputation scores and those of their rivals, as well as their adjusted payoffs in the given round.

#### (iii) Manipulation 1: competition for reputation

Above the PDs, reputation scores played a role for the payoffs in phase II. Payoffs were adjusted according to the reputation score players received on average relative to a reference group of five participants (rivals). By introducing small rival groups, we tested whether players try to wreck rivals' reputation by dishonest negative gossip. Rivals were selected randomly at the very beginning of phase II, and they remained the same until the end of the experiment.

A deviance of the participant's mean reputation score relative to the rivals' decreased/increased the participant's payoff. Payoffs from the PDs have not been altered for those who received the same score on average as their rivals. The magnitude of the alteration was determined by the strength of competition (high versus low). One-unit deviance reduced/increased payoffs by HUF 20 (approx. 5.5 euro cents) in high competition and by HUF 2 (approx. 0.55 euro cents) in low competition. Thus, manipulation 1 modified the strength of the competition for reputation scores.

#### (iv) Manipulation 2: mechanisms that can maintain the credibility of gossip

*Cross-checking.* In the cross-checking condition, we allowed players to ask for a second gossip about the same target (see the top row in figure 1). Cross-checking gossip about potential partners could lead to a more reliable assessment of others' willingness to cooperate. In the control condition, the second gossip could be applied to a new target.

*Social bonding*. In the social bonding condition, we manipulated whether gossip could be reciprocated. We analysed the effect of this affiliative action on the reliability of gossip, reputations and cooperation. In each round, players could send two messages in a row to a pre-designated receiver. In the social bonding manipulation, the second gossip could be reciprocated to the source of the first gossip (see the bottom row in figure 1). In the control treatment, the receiver of the second message was a new subject (see the middle row in figure 1). We consider this reciprocated action as a less costly opportunity for bonding before participants face a more conflicted situation in the next round's PD game (see matching of next PD partners in figure 2).

## 3. Results

### (a) Descriptive statistics

#### (i) Cooperation

**Figure 3.**
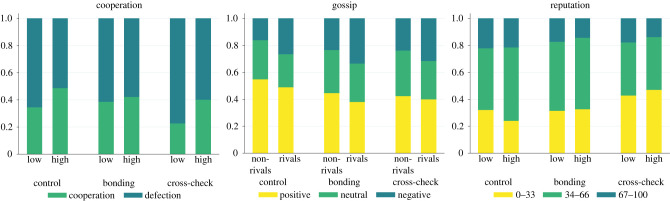
Cooperation, the valence of the gossip and trust by manipulations. Cooperation is higher in high-competition treatments. Negative gossip is more prevalent when rivals are the targets of gossip. Reputation scores are lower in cross-checking treatments. (Online version in colour.)

Baseline cooperation without communication in the first five rounds (38.7%) has increased in round 6, after the introduction of gossip and the opportunity for reputation building (52.1%). Afterwards, cooperation eroded gradually till the last round of the experiment (29.6%). High competition induced an average level of 43.7% cooperation, while the cooperation rate in the low competition was 31.7%. Cross-checking generated an average cooperation rate of 30.9%, while social bonding produced an average cooperation rate of 40.3% similar to the control condition (41.8%), in which neither social bonding nor cross-checking opportunities were present (figure 3).

#### (ii) Gossip

Participants used gossip in 86.7% of their opportunities, both under high (87.0%) and low competition (86.5%), but the exploitation of gossip opportunity varied by treatment conditions (control: 95.4%; social bonding: 90.3%; cross-checking: 74.1%). In both the social bonding and cross-checking conditions, opportunities to send gossip were limited because they depended on the initiation of the gossip partner. In the social bonding condition, a second gossip could only be sent in response to the first gossip if it had been sent (in 91.1% of cases, participants used the first gossip opportunity). In the cross-checking treatment, participants could only send the second gossip if they received a request for cross-checking (68.4% of possible requests were sent) given that there was any first gossip to verify (the first gossip was sent in 91.8% of cases). On average, gossip was mainly positive (46.3%), less often neutral (30.2%) or negative (23.4%).

#### (iii) Reputation

Reputation scores (measured on a 100-point scale) did not differ considerably between treatments with low (42.2) and high competition (42.6). Reputation scores reached their lowest average value in the cross-checking (37.5) and in the social bonding condition (41.8), while the average value in the control condition was slightly lower than the initial score of 50 (47.9). In the following sections, we provide insights into the micro-level mechanisms that are responsible for these patterns at the macro level.

### (b) Multilevel mixed-effects models

For the establishment of reputation-based cooperation mediated by second-hand information, such as gossip, three associations are quintessential. First, gossip should be honest, such that it reflects past behaviour. Second, gossip should be believed by the receiver and incorporated into the receivers' perception of the target. Third, receivers have to make decisions according to this cognitive image when they decide about cooperation or defection against the target.

Competition can induce distortion in the first step by encouraging dishonest gossip about rivals. This can make the entire reputation system unreliable because the distortion impedes subsequent associations. If second-hand information or bonding considerations between the sender and the receiver do not provide guidance to make appropriate decisions, individuals will rather choose defection as a secure strategy that may result in the collapse of cooperation. In the following, we examine the presence of dishonesty, its potential escalation by competition, and whether social bonding and cross-checking can correct this distortion. Applying mixed effect multilevel models, we adjust our analysis to individual's repeatedly observed decisions.

#### (i) Reliability of gossip

Using multilevel ordered logistic models, we found that, regardless of all other factors, gossip about rivals was more negative (*β* = −0.29, *p* < 0.001, model 1; electronic supplementary material, table S1) suggesting that players tried to improve their own position to the detriment of rivals. When the competition was low, in the cross-checking condition, we did not detect any dishonesty about rivals (*β* = 0.42, *p* < 0.05, model 2; electronic supplementary material, table S1), which means that the opportunity for cross-checking significantly holds back negative gossip about rivals compared to the control condition. This apparent counterforce disappeared in high competition (*β* = −0.86, *p* < 0.01, model 2; electronic supplementary material, table S1), despite the fact that dishonesty has not been intensified by competition (*β* = 0.27, *p* = 0.18, model 2; electronic supplementary material, table S1).

Apart from the distortion created by rivalry, gossip was sent in an honest way in the sense that it was aligned with targets' PD decisions (if sender was an involved PD partner in a given round): if the target defected, then gossip was less positive (*β* = −1.36, *p* < 0.001, model 2; electronic supplementary material, table S1); while if the target of gossip cooperated with the sender, then gossip was more positive (*β* = 1.35, *p* < 0.001, model 2; electronic supplementary material, table S1).

Since senders were not always in direct encounters with gossip targets, gossip could rest on players’ private reputation assessment as well. The higher the target's reputation was, the more likely a positive message was sent about that person (*β* = 0.03, *p* < 0.001, model 2; electronic supplementary material, table S1). Compared to the control group, in the cross-checking treatment, the likelihood of sending positive gossip increased less steeply as the reputation score increased (*β* = −0.01, *p* < 0.001, model 2; electronic supplementary material, table S2). In other words, gossip about players with good reputations was less positive in this treatment.

#### (ii) Building a reputation system on believed information

Being aware of the presence of dishonesty, we examine whether gossip was believed and was incorporated into private reputation assessments. Participants privately assigned reputation scores to others, to preserve and be able to recall their previous behaviours. When doing so, they potentially integrated evaluations received from others into their scores. Participants modified their evaluations in line with the gossip they received. Positive messages increased (*β* = 7.42, *p* < 0.001, model 1; electronic supplementary material, table S3), negative messages decreased (*β* = −5.14, *p* < 0.001, model 1; electronic supplementary material, table S3) the allocated reputation scores to the target compared to those about whom neutral gossip have been heard. There were differences between the manipulations with regard to how messages had been incorporated into reputation ratings. In high competition, negative messages decreased reputations with a larger magnitude (*β* = −2.53, *p* < 0.05, model 3; electronic supplementary material, table S3) and positive messages were less rewarding (*β* = −2.49, *p* < 0.01, model 3; electronic supplementary material, table S3). In the social bonding condition, positive messages increased targets' reputation scores more than in the control condition (*β* = 4.67, *p* < 0.001, model 2; electronic supplementary material, table S3).

We note that the trustworthiness of the gossip source played a role in accepting gossip as true. No credit was given to negative messages when the source of gossip had a bad reputation (*β* = −2.22, *p* = 0.06, model 2; electronic supplementary material, table S4). Moreover, the penalty for negative gossip increased as the reputation of the sender improved (*β* = −0.05, *p* < 0.05, model 2; electronic supplementary material, table S4). Besides gossip, as expected, reputations were formed by participants’ direct experience as an involved party in the PD: assigned reputation scores were adjusted in the positive direction after cooperation (*β* = 8.63, *p* < 0.001, model 1; electronic supplementary material, table S3), and in the negative direction after defection by the interaction partner (*β* = −9.06, *p* < 0.001, model 1; electronic supplementary material, table S3).

Apart from first- and second-hand information, two other factors affected participants' assessments. Participants appreciated the gossip they received: gossip senders received slightly better reputation scores (*β* = 0.59, *p* < 0.05, model 1; electronic supplementary material, table S3), and those who could gossip but did not send any messages received lower ratings (*β* = −3.86, *p* < 0.001, model 1; electronic supplementary material, table S3). Also, reputation scores assigned to rivals were significantly lower (*β* = −2.05, *p* < 0.001, model 1; electronic supplementary material, table S3), even if scores from rivals did not affect individuals’ payoff.

#### (iii) Reputation-based cooperation

Regarding the third link of the main narrative, we found evidence that cooperation was conditional on the reputation scores of PD partners (*β* = 0.02, *p* < 0.001, model 1; electronic supplementary material, table S5). From the manipulations, only high competition led to a higher level of cooperation regardless of the partner's reputation (participants cooperated more even if their partners had a bad reputation; *β* = 1.42, *p* < 0.001, model 2; electronic supplementary material, table S5), but the positive impact of reputation scores was weaker in this treatment (*β* = −0.01, *p* < 0.001, model 2; electronic supplementary material, table S5) and the likelihood of cooperation with trustworthy individuals returned to the level of treatments with low competition. The positive effect of strong competition kept the otherwise declining cooperation (*β* = −0.11, *p* < 0.001, model 2; electronic supplementary material, table S6) at a higher level over time (*β* = 0.06, *p* < 0.001, model 2; electronic supplementary material, table S6).

#### (iv) Overall reflectivity

Finally, we provide an overview of whether a reliable reputation system has been established by honest gossip, gossip-based trust formation and reputation-based cooperation. As a result of these links, a reliable reputation system can develop that reflects past actions well; thus it provides a good guide for individuals to conditionally cooperate. Surprisingly, despite dishonest gossip about rivals, the reputation system helped subjects to make good decisions in each condition: the more someone cooperated in previous rounds, the more likely others cooperated with that person (*β* = 1.25, *p* < 0.001, model 1; electronic supplementary material, table S7). The overall association did not differ between conditions (see models 2, 3, 4; electronic supplementary material, table S7). Even if we see differences in the strengths of the operating mechanisms between conditions, we observed a good overall efficiency of the reputation system in our experiment.

## 4. Conclusion

A reputation system can effectively maintain cooperation only if it is based on reliable information spreading. Gossip—an evaluative communication about third parties—could be the channel of reliable information transmission and hence could contribute to the maintenance of cooperation ([79] for review). There is significant doubt, however, about why gossip should be honest and reliable at all [80]. In this study, we investigated mechanisms that could alter whether gossip could be a successful informal mechanism that establishes cooperation through the construction of reliable reputations.

First, we argued that strong direct rivalry for reputations could increase opportunistic use of gossip and hence decrease the reliability of the information received. We have designed the high-competition condition in our experiment in a way that direct rivalry with a set of other participants meant a distribution of monetary payoffs depending on *relative* reputations. Second, we argued that once the opportunity is given, individuals actively seek and cross-check social information to condition their future actions on a better-informed ground, which improves the reliability of reputations they assign to others. While not just sending, but also seeking gossip possibly takes place in complex ways in human interactions, we implemented cross-checking as a single opportunity to ask a second opinion about the same target. Third, we argued that social bonding motives could increase the credibility of social information exchange and hence make reputations reliable. Although it was not possible to create real social bonds between participants in the experiment, we selected a single characteristic that is typical of social bonding and friendship formation and could also be introduced in an abstract experimental setting: *reciprocity in communication*. Note that reciprocity in communication did not mean reciprocity in interactions as participants played PD games against different partners to follow the settings described in models of indirect reciprocity [5–8]. We expected that both cross-checking and social bonding operationalized as reciprocity in communication between the sender and the receiver could be efficient mechanisms ensuring honesty of gossip in conditions of intense competition for reputations.

Even if gossip and reputation scores were mutually aligned with each other and with the PD decisions, cooperation did not emerge to a very high rate in any of the conditions. Competition for reputations had divergent effects in our experiment. On the one hand, messages about rivals were more negative, which diminished the reliability of assigned reputations. On the other hand, cooperation was affected positively by the strength of competition. In line with competitive helping theory, rivalry increased cooperation regardless of the reputation of partners (see [81]). Still, no escalation of cooperation was observed; only the decline of cooperation slowed down (cf. [82]).

Though reputation scores grew more as a result of positive messages received, the possibility of social bonding did not cause significant improvement for cooperation. Our results are consistent with the fact that people place more weight on positive information if it comes from a stronger social bond [83]. The integration of received information from trusted sources is important for a well-functioning reputation system, but as social bonding did not improve significantly how reputations are used to condition behaviour, this treatment did not substantially improve cooperation overall.

In the cross-checking condition, we observed a greater cautiousness of participants. Participants were less courageous in sending positive gossip about trustworthy partners. Besides greater cautiousness, participants often received conflicting information about the same target (see electronic supplementary material, table S8), which may lower the reliability of communication even compared to no information [84]. Mixed gossip could have an averaging [20] and a majority effect [85] on reputations. Surprisingly, people inclined to doubt multiple negative opinions as well [20,50] (see *β* = 0.96, *p* = 0.62, model 1; electronic supplementary material, table S9).

Participants in the cross-checking and social bonding conditions were assigned lower reputation scores in general. Lower reputation scores in these conditions—measured as trustworthiness—may have been caused by a general lack of trust caused by the inefficient [86] and sometimes contradicting information participants received. Social information needs to be available in large amounts to assist cooperation [14,87]. Correspondingly, the reputation of gossip sources was eroded if they failed to provide information.

Confidence in gossip from trustworthy sources was higher [84,88]. People seek information from sources considered trusted [71], probably because of their (perceived) good access to information. Therefore, gossip and the dynamics of reputation and cooperation should be considered from the perspective of the social network structure and the position of relevant individuals within (see [89] for review, [90]).

Our results suggest that a reliable reputation system is not a sufficient condition for cooperation in situations of moderate conflict of interest. At the same time, we found that relative competition seems to play an important role for cooperation, which could be linked with keeping up with others (*loss avoidance*) or achieving reputational benefits (*status maximization*) for the development of widespread human cooperation [34,35,38,41,91–93].

Overall, while we found effects of intensified competition, cross-checking and social bonding for the reliability of gossip, building up of reputations, and partly on conditional behaviour, none of these mechanisms in their abstract form and out of social context were able to sustain a high level of cooperation in the laboratory. Note that gossip was implemented in a very simplified form, as transmission of evaluative social information (sending an emoticon) about the target. This certainly limits the generalizability of our results to empirical situations in which the power of gossip is enhanced in extensive communication.

## Data accessibility

The data are provided in electronic supplementary material [94].
